# Chromosome-level genome assembly and population genomics reveals crucial selection for subgynoecy development in chieh-qua

**DOI:** 10.1093/hr/uhae113

**Published:** 2024-04-22

**Authors:** Min Wang, Zhenqiang Cao, Biao Jiang, Kejian Wang, Dasen Xie, Lin Chen, Shaoqi Shi, Songguang Yang, Hongwei Lu, Qingwu Peng

**Affiliations:** Vegetable Research Institute, Guangdong Academy of Agricultural Sciences, Guangdong Key Laboratory for New Technology Research of Vegetables, Guangzhou 510640, China; Vegetable Research Institute, Guangdong Academy of Agricultural Sciences, Guangdong Key Laboratory for New Technology Research of Vegetables, Guangzhou 510640, China; Vegetable Research Institute, Guangdong Academy of Agricultural Sciences, Guangdong Key Laboratory for New Technology Research of Vegetables, Guangzhou 510640, China; State Key Laboratory of Rice Biology and Breeding, China National Rice Research Institute, Chinese Academy of Agricultural Sciences, Hangzhou 310012, China; Vegetable Research Institute, Guangdong Academy of Agricultural Sciences, Guangdong Key Laboratory for New Technology Research of Vegetables, Guangzhou 510640, China; Vegetable Research Institute, Guangdong Academy of Agricultural Sciences, Guangdong Key Laboratory for New Technology Research of Vegetables, Guangzhou 510640, China; Vegetable Research Institute, Guangdong Academy of Agricultural Sciences, Guangdong Key Laboratory for New Technology Research of Vegetables, Guangzhou 510640, China; Vegetable Research Institute, Guangdong Academy of Agricultural Sciences, Guangdong Key Laboratory for New Technology Research of Vegetables, Guangzhou 510640, China; State Key Laboratory of Rice Biology and Breeding, China National Rice Research Institute, Chinese Academy of Agricultural Sciences, Hangzhou 310012, China; Vegetable Research Institute, Guangdong Academy of Agricultural Sciences, Guangdong Key Laboratory for New Technology Research of Vegetables, Guangzhou 510640, China

## Abstract

Chieh-qua is an important cucurbit crop and very popular in South China and Southeast Asia. Despite its significance, its genetic basis and domestication history are unclear. In this study, we have successfully generated a chromosome-level reference genome assembly for the chieh-qua ‘A36’ using a hybrid assembly strategy that combines PacBio long reads and Illumina short reads. The assembled genome of chieh-qua is approximately 953.3 Mb in size and is organized into 12 chromosomes, with contig N50 of 6.9 Mb and scaffold N50 of 68.2 Mb. Notably, the chieh-qua genome is comparable in size to the wax gourd genome. Through gene prediction analysis, we have identified a total of 24 593 protein-coding genes in the A36 genome. Additionally, approximately 56.6% (539.3 Mb) of the chieh-qua genome consists of repetitive sequences. Comparative genome analysis revealed that chieh-qua and wax gourd are closely related, indicating a close evolutionary relationship between the two species. Population genomic analysis, employing 129 chieh-qua accessions and 146 wax gourd accessions, demonstrated that chieh-qua exhibits greater genetic diversity compared to wax gourd. We also employed the GWAS method to identify related QTLs associated with subgynoecy, an interested and important trait in chieh-qua. The *MYB59* (*BhiCQ0880026447*) exhibited relatively high expression levels in the shoot apex of four subgynoecious varieties compared with monoecious varieties. Overall, this research provides insights into the domestication history of chieh-qua and offers valuable genomic resources for further molecular research.

## Introduction

Chieh-qua (*Benincasa hispida* Cogn. var. Chieh-qua How) (2n = 2x = 24), also known as hairy melon, fuzzy melon, or moqua [1], belongs to genus *Benincasa* in the plant family Cucurbitaceae, renowned for cucurbits and gourds. As an economically important and characteristic vegetable, chieh-qua has been planted in South China over 300 years and is now widely cultivated in India, Malaysia, and many other Southeast Asian countries [2]. Generally, the immature fruit of chieh-qua are considered as a commodity, with many bristle-like trichomes on its epidemis [3]. An ancient Chinese medical book, ‘Materia Medica for Original’, recorded that chieh-qua possessed the function of a wax gourd; however, it is warm in nature and beneficial for the human stomach, spleen, and intestines. Its nutritive value and the use of chieh-qua in traditional herbal remedies was also recorded in the compendium of material medica (Ben Cao Gang Mu in Chinese) by Li Shizhen, a noted traditional Chinese medicine specialist in the Ming Dynasty.

Since the first *Cucurbitaceae* ‘Chinese long’ 9930 (*Cucumis sativus*) genome in 2009 was sequenced [4], several cucurbits such as other cucumber materials [5, 6], watermelon (*Citrullus lanatus*) [7, 8], melon (*Cucumis melo*) [9–11], bottle gourd (*Lagenaria siceraria*) [12, 13], wax gourd (*B. hispida*) [14, 15], bitter gourd [16, 17], snake gourd (*Trichosanthes anguina*) [18], chayote (*Sechium edule*) [19] and pumpkin, squashes, and gourds (*Cucurbita maxima* and *Cucurbita moschata*) [20, 21] have been successfully assembled, which provided crucial genome resources for the genetic breeding improvement of cucurbits. However, knowledge of chieh-qua genetics and the genome is currently absent. Chieh-qua is like a variety of wax gourd, while there are also some differences between them such as the original area, commercial fruit types, and sex differentiations. Thus, the assembled wax gourd genomes could not really be used in chieh-qua studies. Currently, only transcriptome sequences for seedling leaves have been developed for chieh-qua [22, 23]. Hence, the sequence and assembly of the chieh-qua genome are necessary to investigate the genome differences from the wax gourd and explore the diversity of its genetic basis.

**Figure 1:**
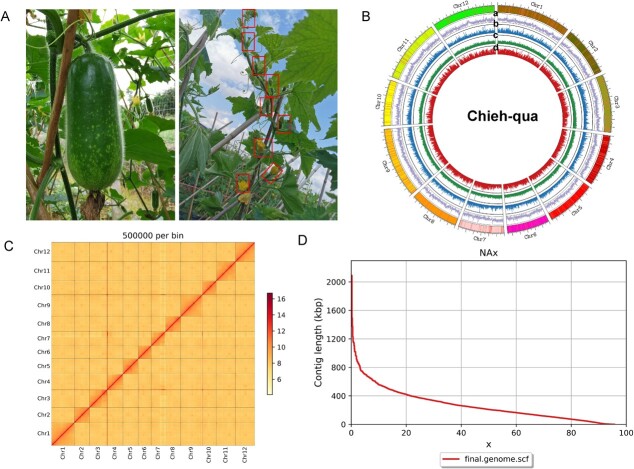
Overview of the chromosome-level genome of chieh-qua. **A** The fruit of chieh-qua ‘A36’ (left) and the female flowers at top main stem (right). **B** Overview of the chieh-qua genome: a, GC content; b, repeat sequence coverage; c, gene density; d, noncoding RNA density. **C** Hi-C heat map of chromosome interactions. **D** The contig length corresponding to genome.

Plant sex expression is determined by the formation and distributions of female, male, and hermaphroditic flowers [24]. The cucurbit family have become model systems for sex determination studies, as they have all seven sex forms such as monoecious, gynoecious, androecious and so on [25]. As a continuously harvested vegetable, gynoecy or subgynoecy of chieh-qua exert crucial roles in producing a higher yield and increased efficiency of hybrid seed production. The cucurbit has formed two pathways for the regulation of sex determination including the *WIP1* transcription factor pathway [26] and ethylene synthesis-related genes pathway [27], especially in melon and cucumber. In chieh-qua, the *CqNET4*, which encodes the networked protein, was likely the gynoecious candidate gene, different from the reported gynoecious genes [28]. However, no crucial genes related to subgynoecy have been reported in chieh-qua.

In this study, we began by sequencing and assembling the genome of chieh-qua ‘A36’ using a combination of PacBio long reads, Nanopore sequencing, and Hi-C chromatin interaction maps. Subsequently, we resequenced the genomes of 129 distinct chieh-qua accessions and conducted population genomic analysis and GWAS to investigate the subgynoecious traits. The assembled genome and the observed population genetic diversity offer valuable resources for genomics research and contribute to our understanding of subgynoecy modification and genetic breeding in chieh-qua.

## Results

### ‘A36’ genome assembly and evaluation

In this study, a relatively high-quality genome of the chieh-qua high generation inbred line ‘A36’ was assembled using a hybrid method including Illumina HiSeq, PacBio, and Hi-C strategies. ‘A36’ is a Jiangxin type chieh-qua, exhibits important agronomic traits such as high female node rate, high yield, good quality (Fig. 1A), which is wildly popular in South China and Southeast Asia. For the preliminary identification of ‘A36’ chieh-qua, we performed next generation sequencing and K-mer analysis to estimate its genome size and heterozygosity. In order to acquire a chromosome-level reference genome of chieh-qua, we approximately obtained a total of 98.2 Gb of raw NGS data. After data quality control (QC), we achieved 98.1 Gb of clean data, which represents about 107× sequencing depth. Using the third-generation sequencing technology of the Pacbio Sequell platform, a total of 164 Gb long reads were generated and assembled to 952.5 Mb genome sequence with 765 contigs, and the contig N50 is 6.9 Mb. Then, the Hi-C technology was combined to evaluate the interactions of chromosomes in 3D-DNA. Ultimately, the final chieh-qua ‘A36’ genome was 953.3 Mb anchored to 12 chromosomes with 68.2 Mb for scaffold N50 (Table 1), which had an overview of the genome assembly (Fig. 1B). By using the Hi-C paired-end reads to anchor the contigs, we obtained a heat map of Hi-C assembled chromosomes, confirming the completeness of the genome assembly (Fig. 1C and D).

**Table 1:** Statistics of genome sequencing, Hi–C assembly and gene assessment in BUSCO

**Parameter**	**Value**
***Nanopore sequencing***	
Contig number	765
Contig N50 (Mb)	6.9
Genome size (Mb)	952.5
***Hi–C assembly***	
Scaffold number	1515
Scaffold N50 (Mb)	68.2
Genome size (Mb)	953.3
***BUSCO evaluation***	
Complete BUSCOs	96.1% (1552)
Complete and single-copy BUSCOs	94.5% (1526)
Complete and duplicated BUSCOs	1.6% (26)
Fragmented BUSCOs	1.4% (23)
Missing BUSCOs	2.5% (39)
Total lineage BUSCOs	1614

For assessing the integrity of ‘A36’ genome integrity, we used the BUSCO database 5.3.1 [29] with e-value <1e^−5^. Results showed the total lineage BUSCOs were 1614, which included 1552 (96.1%) complete BUSCOs (Table 1), which was relatively complete for the chieh-qua genome assembly including 94.5% single copy, 1.6% duplicated copy, 1.4% fragmented, and 2.5% missing BUSCOs (Table 1).

### Genome annotation

A comprehensive pipeline from the combined evidence of ab initio prediction, protein homologs, and transcriptome sequencing data were used for genome annotation. In total, repetitive sequences (623.80 Mb) made up 65.43% of the whole genome with the long terminal repeats (LTRs) accounting for the highest ratio (55.9%) among the chieh-qua genome. Two LTR subtypes, Copia-LTRs (175.93 Mb) and Gypsy-LTRs (337.71 Mb) represented 18.45% and 35.42% of the whole genome (Table S1, see online supplementary material), respectively. The genome annotation pipeline predicted the genetic structure and 34 082 protein-coding genes and chieh-qua were detected (Table 1). The max gene length was 135.1 kb, while the min was 150 bp. The median and average of gene lengths were 1501 bp and 3020 bp, respectively (Table S2, see online supplementary material). A total of 24 953 (72.2%) genes were annotated, while 9489 genes unannotated and 18 327 InterPro and 13 618 GO were obtained, respectively (Tables S3 and S4, see online supplementary material). By BUSCO analysis of the genome annotation, we also found that the whole genome annotation was complete (94.9%) with 94.2% single copy, 0.7% duplicated copy, 2.5% fragmented, and 2.6% missing (Table S5, see online supplementary material).

### Comparative genomics analysis

**Figure 2:**
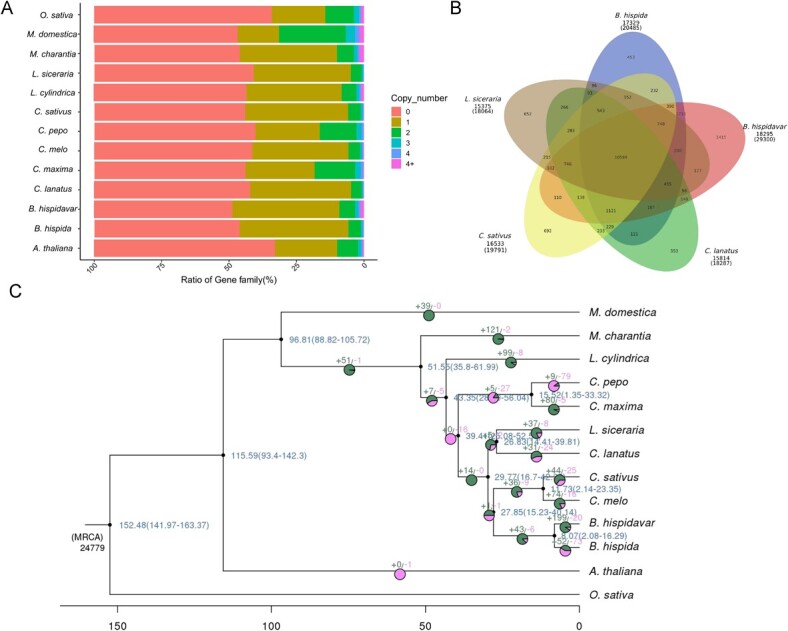
Analysis of gene families and phylogeny of chieh-qua and other plant species genomes. **A** Gene copy number distribution in chieh-qua and other 12 plant genomes. **B** Venn diagram in chieh-qua and four related plants (*Benincasa hispida*, *Lagenaria siceraria*, *Citrullus lanatus*, *Cucumis sativus*). **C** Species phylogenetic tree and expansion and contraction of gene family from chieh-qua and 12 gene families. Green and red colors indicate the gene family expansions and contractions on different species tips and lineages, respectively.

To understand the evolution of the chieh-qua genome, except to chieh-qua (*B. hispidavar*), a total of 12 selected plant species were selected for the verification of homologous genes, the clustering analysis of gene family, and the enrichment of single and multiple copy genes, which included the *B. hispida*, *C. lanatus*, *C. maxima*, *C. pepo, C. sativus*, *L. cylindrica*, *L. siceraria*, *M. charantia*, *M. domestica*, *O. sativa*, *A. thaliana*. A toal of 47 325 gene families were analysed, representing 2395 common gene families represented, or 967 specific gene families in the chieh-qua genome (Fig. S1A, see online supplementary material). Next, we performed the orthogroup gene statistics and chieh-qua contained 43.7% one-copy genes, which is lower than wax gourd (63.3%) and cucumber (62.9%) and 12.8% two-copy genes and 22.4% four-plus-copy genes, which higher than other species (Fig. 2A). Cluster analysis of gene families was performed in chieh-qua, wax gourd, cucumber, watermelon, bottle gourd, and indicated the number of chieh gene families (18 295) was higher than the other four species (Fig. 2B).

The phylogenetic tree with molecular dating and gene family expansions/contractions were analysed based on a number of 1293 single-copy protein sequences in these plant species. The results showed that chieh-qua was the closest to wax gourd in all of the species examined (Fig. 2C). Chieh-qua and wax gourd diverged from their common ancestor approximately 2.08–16.29 million years ago (Fig. 2C; Fig. S1B, see online supplementary material). In chieh-qua, there were 199 and 20 gene families underwent significant expansion and contraction, respectively, differing from wax gourd (52 genes expansion, 73 genes contraction) (Fig. 2C). Gene Ontology (GO) and Kyoto Encyclopedia of Genes and Genomes (KEGG) enrichment were used to analyse the specific gene families in chieh-qua and showed that the unique genes were related to ribosome, oxidative phosphorylation, ABC transporters, and RNA polymerase (Fig. S2A and B, see online supplementary material).

### Basic population genetic characteristics analysis

**Figure 3:**
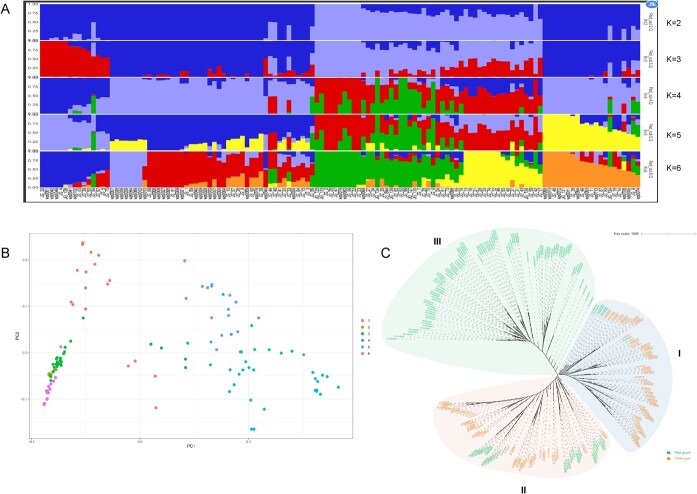
Analysis of the population evolutionary of chieh-qua and wax gourd. **A** Population structure of clusters number (K = 2–6). The *x*-axis indicates different chieh-qua accessions, and the left *y*-axis quantifies cluster membership. **B** Principal component analysis (PCA) plots of chieh-qua populations. **C** Evolutionary analysis of chieh-qua and wax gourd accessions.

To understand the genomic variation of multi-chieh-qua varieties, we re-sequenced the 129 chieh-qua accessions with an average sequencing depth of 11× from four southern provinces in China (Guangdong, Guangxi, Fujian, and Sichuan) (Fig. S3, see online supplementary material). A total of 9419 million clean reads were yielded, among them, 98.26% were mapped to the assembled ‘A36’ genome. After filtering, we identified 4 324 889 high-quality SNP loci (minor allele frequency >5%), which were used for the population genetic analyses. In order to select the proper K value, we caculated the cross-validation error (CV error) of K from1 to 9 (Fig. S4A). To better know the chieh-qua genetic background, we performed population structure analysis with ancestral group values (K) ranging from 2 to 6 based on high-quality SNPs. Fig. 3A (Fig. 3B; Table S6, see online supplementary material). We also found that the I and IV subgroups had relatively more individuals, with 36 and 32, respectively. The I subgroup accessions mainly belonged to ‘Jiangxin Type’, while the V subgroup accessions mainly belonged to ‘Heimao Type’ (Table S6, see online supplementary material). Next, in order to understand the evolutionary relationship of chieh-qua and wax gourd, we performed phylogenetic analysis using SNPs from 129 accessions and the reported 146 wax gourd accessions (including 10 chieh-qua accession) [14]. Combing the Assemblitics sofeware, the genes structural variations of chieh-qua and wax gourd were analyzed, and the length distribution of different structural variation types were shown in the figure (Fig. S4B), respectively. A total of 275 accessions were divided into three genetic groups: I (86 accessions with only seven wax gourd accessions), II (99 accessions with 60 chieh-qua accessions), and III (90 accessions without chieh-qua accessions) (Fig. 3C). By LD decay analysis, we found that the r^2^ values in chieh-qua and wax gourd populations were both about 0.8, and as the distance became longer, the linkage degree of chieh-qua was a little higher than wax gourd (Fig. S5A, see online supplementary material).

**Figure 4:**
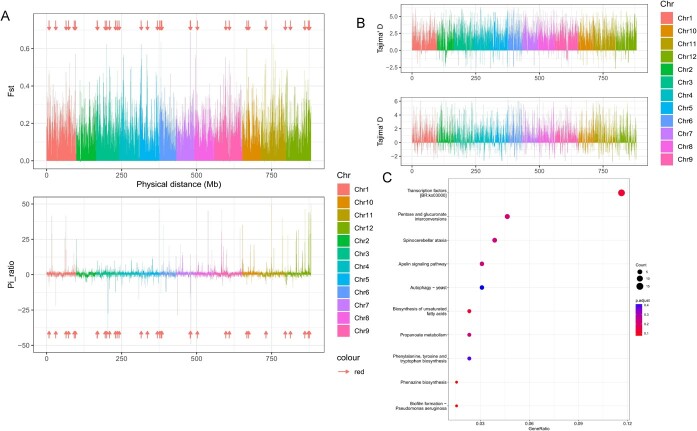
Analysis of selective sweeps in chieh-qua and wax gourd. **A** The distribution of selective sweeps between chieh-qua and wax gourd. Red vertical boxes represent the selective sweeps. **B** Tajima’D analysis of the selected genomic regions between chieh-qua and wax gourd. **C** KEGG analysis of related genes in the selective sweeps.

As chieh-qua is a variety of wax gourd, in order to get to know the population selective stress, we also combined the previous sequence data of wax gourd to perform statistical analysis of the Pi and Fst value among chieh-qua and wax gourd populations. We obtained 228 of the differentiation zone by combing the adjacent interval based on the Fst over 0.31. According to the absolute value of Pi_ration over 4.217, a total of 178 differential selection sections were acquired; among them, 135 sections were strongly selected in wax gourd, 43 sections were strongly selected in chieh-qua (Fig. 4A). Combining the differentiation zone and selection sections together, 33 common regions were finally obtained including 14 sections in wax gourd and 19 sections in chieh-qua and the selective regions were mainly located in Chr.1 and Chr.3 and there were no selected regions in Chr.2 and Chr.4 (Fig. 4A; Table S7, see online supplementary material). In order to deeply analyse the genomic region with key roles in the population differentiation of chieh-qua and wax gourd, the Tajima’s D was used in our study. Results showed that there were more selected genomic regions in wax gourd (454 regions with 193 Mb) than chieh-qua (179 regions with 63.8 Mb) (Fig. 4B). Among them, the length of more than 1.0 Mb areas: there are five and 12 in chieh-qua and wax gourd, respectively, of which four areas were shared between the two groups (Fig. 4B), indicating more genetic purification options might be involved in the population evolution of wax gourd than chieh-qua, which is similar to those of pi value analysis. To better get to know the gene classification, we performed KEGG analysis and found that genes in the selection region were mainly related to the transcription factors, pentose and glucuronate interconversions, spinocerebellar ataxia (Fig. 4B; Table S8, see online supplementary material).

### Identification of genes or loci related to subgynoecy

**Figure 5:**
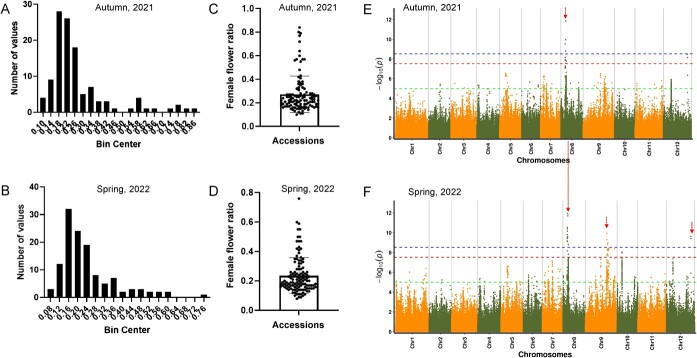
GWAS mapping associated with chieh-qua subgynoecy. **A, B** The frequency distribution of subgynoecious trait in different accessions in autumn 2021 (**A**) and spring 2022 (**B**). **C, D** Analysis of box plot for subgynoecy from the two seasons. **E, F** Manhattan plot summarizing the GWAS results of subgynoecy for twice using the EMMAX method. Red vertical lines indicate the overlapping region of the trait from two seasons as highlighted below.

Subgynoecy exerts a better advantage on high female flower rate and high yield. As of the time of writing, a few genes underlying subgynoecy have been isolated in chieh-qua. To explore the GWAS potential for identifying the causal gene of subgynoecy, we carried out an association research using a panel of 129 diverse accessions (Fig. S4, see online supplementary material) related to the trait in the autumn of 2021 and the spring of 2022 (Table S9, see online supplementary material). To phenotype the subgynoecy trait, the female node rate of the main stem was used to indicate the subgynoecy of each accession. Both the frequency distribution of the subgynoecy obeyed a skewed normal distribution in autumn 2021 (Fig. 5A) and spring 2022 (Fig. 5B) and most of the accessions have a relatively low female node rate in autumn (Fig. 5C) and spring (Fig. 5D).

In order to detect the genomic loci controlling subgynoecy, we first identified a total of 15 associated SNPs (−log10 (*P*) > 8) (Fig. 5E and F; Fig. S5B and C, see online supplementary material) including nine SNPs on Chr.8, four SNPs on Chr.9, and two SNPs on Chr.12 using the GEMMA method. In order to identify the GWAS results, we also employed the FaST-LMM program to detect some associated SNPs and results showed the crucial region was located in the same region in autumn (Fig. S6A and B, see online supplementary material) and spring (Fig. S6C and D, see online supplementary material). However, only six significant associated signals were detected in autumn with four SNPs on Chr.9 and two SNPs on Chr.12. Among the total 15 signals of spring and autumn, three SNPs named *CqSg8.1*, *CqSg8.2* and *CqSg8.3* overlapped during the two seasons (Fig. 5E and F). Thus, we mainly focused on these three loci and further found that the *CqSg8.1* (14413603), *CqSg8.2* (14554474), *CqSg8.3* (14645233) were quite near the four genes (*BhiCQ0880026444, BhiCQ0880026445, BhiCQ0880026446, BhiCQ0880026447*) in the 231.6-kb region (14 413 603 ~ 14 645 233). In view of this, we extended the candidate interval by 50 kb, which became 331.6 kb, and a summary of 10 genes (*BhiCQ0880026440-BhiCQ0880026449*) were identified as the impossible candidate genes associated with subgynoecy.

Next, a BSA assay was performed using the F2 genetic population from JG1 (subgynoecy) and JG128 (monoecy) (Fig. S7A, see online supplementary material). The female flower ratio of JG1 was about 70% in spring 2022 and autumn on average, while the JG128 was about 10%. The F1 female flower ratio was about 16% on average (Fig. S7B, see online supplementary material), and their F2 population exhibited the skewed normal distribution in autumn 2022 (Fig. S7C, see online supplementary material), indicating that subgynoecy was a quantitative trait. The two subgynoecy-related pool (female flower ratio over 45%) and monoecy-associated pool (female flower ratio below 12%) were then constructed, plus parents, to the performed BSA sequence. After filtering raw data, we finally obtained 201 703 862 and 213 442 458 reads from the subgynoecy pool and monoecy pool, respectively. An average Q20 value of 96.62%, and an average total mapped of 99.36% were obtained (Table S10, see online supplementary material). Then, based on these different SNP callings, we performed BSA for identifying the main locus related to subgynoecy. We found that the related locus was located on a 4.9-Mb region on Chr.8 from 12 935 829 to 17 928 461 bp via the ED algorithm (Fig. S7D, see online supplementary material) and combining the SNP index algorithm (Fig. S7E, see online supplementary material), whose region also overlapped. In order to narrow the BSA-seq detecting region, SSR and Indel markers were employed in the F2 population. However, we finally narrowed it into 1.3 Mb flanked with Indel-3 and SSR08 markers (Fig. S7F, see online supplementary material) as no recombinant single plants existed in the F2 population. During the region, a total of 28 genes exhibited nonsynonymous SNV, which included the four loci of GWAS.

### Expression analysis of candidate genes related to subgynoecy

**Figure 6:**
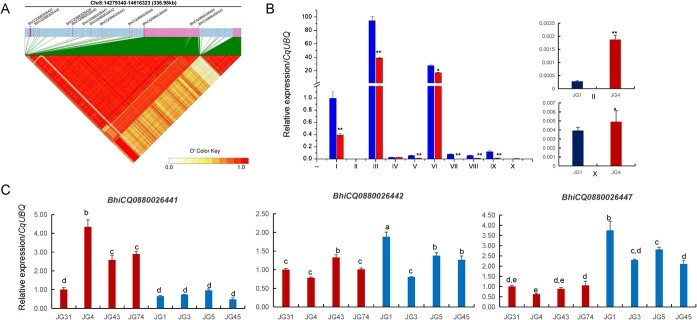
Expression analysis of candidate genes associated with subgynoecy. **A** LD heat map surrounding the candidate locis peak. **B** qRT-PCR analysis of 10 candidate genes between subgynoecy (JG1) and monoeciy (JG4). **C** Three genes expression of the shoot apex among each of four subgynoecious and monoecious materials. Data are presented as the means ± SDs (*n* = 3). ^*^ and ^**^ represent significant differences at the 0.05 and 0.01 level, respectively (Student’s *t* test). Different letters indicate significant differences at 0.05 levels.

Given that the potential candidate genes of *CqSg* locus were analysed within the 331.6-kb interval centered on the leading SNPs (chr08:14554474), 10 candidate genes were then identified for the main locus (Fig. 6A). Firstly, we detected the 10 genes’ expression levels between the subgynoecy (JG1) and monoecy (JG4) and found that except *BhiCQ0880026441, BhiCQ0880026443*, and *BhiCQ0880026449*, the other genes had relatively high expression in JG1 compared with JG4,while *BhiCQ0880026441* showed higher expression level in JG4 than JG1, and *BhiCQ0880026443* and *BhiCQ0880026449* had no difference between JG1 and JG4 (Fig. 6B). Next, in order to know whether other different sex types showed the same expression tendency, we randomly selected the four lines from each of subgynoecious and monoecious varieties to measure the transcript levels of these 10 genes. We observed that only *BhiCQ0880026447* exhibited relatively high expression levels in the shoot apex of four subgynoecious varieties compared with monoecious varieties, while other genes showed no consistency of overall expression in the four subgynoecious varieties or monoecious varieties (Fig. 6C; Fig. S8, see online supplementary material). In addition, the genome of *BhiCQ0880026447* was cloned and sequenced in three subgenoecious (JG1, JG3, JG45) and monoecious (JG4, JG31, JG128) plants, respectively. Results showed that a nonsynonymous SNP mutation (location on 145, 497, 63) existed among them. It was ‘A’ in the three subgenoecious plants, while it was ‘T’ in the three monoecious plants and the SNP mutation caused an amino acid change from ‘Met’ to ‘Leu’ (Fig. S9, see online supplementary material). Therefore, the *BhiCQ0880026447* gene, encoding a MYB transcription factor, was the most likely candidate for *CqSg* locus and we then designated this geneas *CqSg*.

## Discussion

Chieh-qua stands out as a special economic and popular vegetable crop in China and Southeast Asia for its abundant nutrition, unique flavor, and medical value. Due to lacking the genomic and biotechnological information of chieh-qua, the molecular breeding of this cultivar has lagged behind other cucurbits crops such as melon, cucumber, and watermelon. In this study, we successfully assembled and comparatively analysed the chieh-qua genome through re-sequencing 129 accessions, revealing a high-quality genome and the evolutionary history of chieh-qua. Using a combination of PacBio, Illumina, and Hi-C strategy, the chromosome-level genome assembly of chieh-qua is 953.3 Mb in size, which is similar to wax gourd [14, 15] and snake gourd [18]. The chieh-qua genome size is larger than other cucurbits, such as melon [10, 31], cucumber [30], watermelon [7, 32], pumpkin [33], bottle gourd [13], zucchini [20], bitter gourd, sponge gourd [34], and chayote [19]. Considering chieh-qua as a variety of wax gourd, we conducted a detailed comparison of their genome sizes. The wax gourd B227 [14] and pf3 [15] genomes were published in 2019 and 2023, respectively. B227 had a large mature fruit (~80 cm in length and over 20 kg in weight) with black peel and its genome size was 913 Mb with contig N50 of 68.5 Kb and scaffold N50 of 3.4 Mb, which showed as incomplete and highly fragmented to some extent [14]. The pf3 had relatively small mature fruit (~30 cm in length and ~ 2.5 kg in weight) with white cuticular wax and its genome size was 975.62 Mb with contig N50 of 2.43 Mb and scaffold N50 of 70.97 Mb, which was a chromosome-level reference wax gourd genome [15]. In our study, the chieh-qua A36 had green glossy peel with some plum blossom spots under its young fruit bottom and it showed ~15 cm in length and ~0.75 kg in weight. The A36 genome (953.3 Mb) is larger than B227 (913 Mb), while slightly smaller than pf3 (975.62 Mb). With a contig N50 of 6.9 Mb and scaffold N50 of 68.2 Mb, and the chromosome-anchored size being 881.8 Mb compared with 859.0 Mb in B227 and 926.05 Mb in pf3, this indicates that chieh-qua genome exerts an improvement over B227 genome in assembly and some similarity with pf3 released previously [14, 15]. In general, we present a chromosome-scale and high-quality genome of chieh-qua, the first report of the chieh-qua assembly genome.

Based on the ‘A36’ genome, the evolution of the chieh-qua genome was analysed and the whole-genome sequences of 129 accessions were obtained, which were classified into six clusters. Xie *et al.* [14] reported that 146 wax gourd accessions could contain wild accessions (W), landraces (l), and cultivated accessions (C1 with fruit wax and C2 without). Among them, five chieh-qua materials belonged to the I cluster. However, no other chieh-qua varieties were selected, limiting the further study of the evolution between chieh-qua and wax gourd. Here, we classified chieh-qua into six groups, more than wax gourd. Combining the 146 and 129 accessions for phylogenetic analysis, they could be classified into three clusters together and only one cluster did not include chieh-qua, hinting that wax gourd had formed its own type during the domestication course. The wild group (5.9 × 10^−3^) had higher π value than other landrace (1.1 × 10^−3^) and cultivated groups (0.4 × 10^−3^) and predicted that a diverse gene pool existed in the wild group [14]. However, in our study, the average π value in chieh-qua (1.4 × 10^−3^) is higher than wax gourd (1.1 × 10^−3^), indicating that the genetic diversity in chieh-qua is more abundant than wax gourd. By Fst and Pi screening, a total of 19 sections were strongly selected in chieh-qua than wax gourd (14 sections), suggesting the key selection process to determine the two varieties mainly occurred in chieh-qua. We randomly analysed a crucial region 20 400 001 ~ 20 840 000 on Chr.10 which was selected in chieh-qua. In the region, the gene *BhiCQ1080004575* encoding ethylene oxidase was selected in chieh-qua. As previous studies [26, 27] reported that genes related to ethylene oxidase were associated with sex determination in cucurbits, we predicted that this gene or the selected region exerted important roles in the sex determination of chieh-qua.

Subgynoecious or gynoecious plants are excellent materials for decreasing the cost of production of hybrids seeds and increasing yields, especially for continuous harvested cucurbits such as cucumber, bitter gourd, and chieh-qua. Although chieh-qua belongs to *Benincasa*, it serves young fruit as a commercial product, which differs from wax gourd, with mature fruit as commodity. Previous studies mainly focused on the molecular regulation of gynoecy and established two pathways containing the recessive *WIP1* and dominant *CsACS1G* paths [26, 27], which are responsible for melon, watermelon, and cucumber gynoecious formation. In addition, by fine mapping strategy, *MC06g0753* (encoding ethylene biosynthesis enzyme) [35] and *CqNET4* (encoding networked protein 4) [28] were predicted as the candidate possible genes in controlling bitter gourd and chieh-qua gynoecy, respectively. However, few studies have reported about subgynoecy in cucurbits as of the time of writing.

In actual production, gynoecious type needs optimal growth conditions to maintain all the female flowers and is tougher to produce self-retaining seeds. Therefore, under regular growth conditions, cultivars with a ration of female flowers might be suitable for cultivation. Subgynoecy could be considered as a special type of monoecy in some extent, which included male flowers at the early growth stage and exclusively female flowers at later growth stages. Nevertheless, due to subgynoecy being influenced by environment, it is challenging to isolate crucial genes in cucurbits. Little research has been reported on detecting crucial QTLs controlling cucumber subgynoecy. Yuan *et al.* [36] reported three QTLs for female flower ratio of cucumber, two located on Chr. 2 and one located on Chr.6. Bu *et al.* [37] detected three subgynocious QTLs: *sg3.1*, *sg6.1*, and *sg6.2*, and a major QTL *sg3.1* was fine mapped to the 799 kb region in cucumber. Another study also revealed three QTLs, and among them, the *sg3.1* and *sg1.1* mainly increase the ratio of female flowers [38]. However, no genes involved in subgynoecy have been identified in other crops until now. At present, our study combined the GWAS method and finally obtained a major *CqSg* locus on Chr.8 in chieh-qua with 10 candidate genes in the three candidate intervals. The *CqSg* locus was not in the selective sweeps for chieh-qua and wax gourd. Among the candidate genes, only *BhiCQ0880026447*, which encoded MYB59, exerts relatively high expression in the shoot apex of those subgynoecious varieties and an SNP mutation existed in three subgynoecious and monoecious plants, respectively, which designated this gene as *CqSg*.

MYB transcription factors exert crucial roles in controlling pathways including plant development, disease resistance, signal transduction, and other biological processes [39, 40], which characterized with conserved DNA-binding domains. A study has reported that EOB2, a member of the R2R3-MYB subgroup, repressed flower bud senescence by inhibiting the production of ethylene [41] and MYBs also function in the stamen development [42–45], ovule fertility [46], as well as pistil length [46, 47]. In our study, only the *MYB59* is relatively up-regulated in the four subgynoecious varieties compared with monoecious varieties, indicating it might play crucial roles in the female flower development. In future, much more molecular research should be performed to identify *MYB59*’s function in subgynoecy formation.

## Materials and methods

### Sample selection and genome sequencing

In order to perform genome sequencing, the total genomic DNA of chieh-qua was extracted, combing a modified CTAB method [48] from chieh-qua seedling leaves of inbred lines ‘A36’ collected from Baiyun base, Guangdong Province. After removing RNA contaminants using RNase A, DNA quality and concentration were detected by a Qubit 3.0 fluorometer (Life Technologies, Carlsbad, CA, USA) and agarose gel electrophoresis. For PacBio sequencing, genomic DNA was fragmented to (CLR: ~20; CCS: ~15) Kb to construct a long-read PacBio SMRT Bell library according to the manufacturer’s instructions (Pacific Biosciences, CA, USA) and sequenced on a PacBio Sequel II platform. Hi-C library was sequenced on the Illumina NovaSeq 6000 platform at 50 × depth based on the manufacturer’s protocols (IIIumina, San Diego,USA). Lastly, we constructed the RNA-seq libraries of ‘A36’ seedling leaves using the True-Seq kit (Illumina) and sequenced on Illumina HiSeq X Ten platform for transcriptome analysis.

### Genome assembly and evaluation

To begin with, the acquired purified Illumina paired-end (PE) reads underwent analysis through k-mer spectrum and heterozygosity level assessment [49] . The *de novo* assembly of PacBio single-molecule real-time (SMRT) reads was conducted utilizing phased assembly graphs with the MaSuRCA assembler (version 3.3.2) to obtain the genomic sequences. The alignment of PacBio and Illumina reads to the genome sequences was performed using minimap2 [50] and SOAP2 [51], respectively. Hi-C sequence data was processed using HiCUP [52], and 3D-DNA [53] was employed to assist in genome assembly, followed by manual refinement using Juicebox (v1.11.08) [54]. Subsequently, the assembled genome underwent evaluation for completeness using the Benchmarking Universal Single Copy Orthologs (BUSCO) version 5.3.1 [29]. The accuracy and integrity of the final ‘A36’ genome assembly were assessed using QUAST version 2.5 [55].

### Repeat annotation and gene prediction in the ‘A36’ genome

We obtained and identified the repetitive sequences and transposable elements within the genome through a combination of *ab initio* and homology-based methodologies, encompassing both the DNA and protein levels. To begin with, the prediction of an *ab initio* repeat library for the genomes was accomplished utilizing LTR_FINDER v1.0.247, along with RepeatModeler (v1.0.3) employing default parameters. Subsequently, this library was aligned with the PGSB Repeat Element Database (http://pgsb.helmholtz-muenchen.de/plant/recat/) to classify the specific type associated with each repeated family. To comprehensively identify the repeats encompassing the entire genome, RepeatMasker (v3.2.9) was employed in conjunction with both the *ab initio* repeat databases and Repbase (http://www.girinst.org/repbase), exploiting the WU-BLASTX search engine. Overlapping transposable elements were compiled and amalgamated. Furthermore, in order to provide comprehensive annotation, we employed the software Tandem Repeats Finder (TRF, v4.04) to identify tandem repeats.

To elucidate the coding potential of genes, we employed three strategies encompassing homology-based inference, *ab initio* prediction, and transcriptomic data integration to unravel the gene architecture within the ‘A36’ genome. The homology-based approach involved aligning protein sequences from SwissProt and various plant species including Arabidopsis, wax gourd, watermelon, cucumber, and melon to the ‘A36’ genome, employing the GenBlastA algorithm [56]. For *ab initio* gene predictions, we relied on the Augustus (v3.2.3) [57] and SNAP (v2006-07-28) [58]. The genome-guided mode was used to assemble RNA-seq reads by Stringtie (v1.3.3b). The three gene model sets were intgerated by EvidenceModeler (v2.1.0). The function annotation of the predicted proteins was performed by InterProScan with default parameters [59].

### Sequence alignment and variation calling

In total, 129 chieh-qua accessions were used, which were obtained by Guangdong Academy of Agricultural Sciences, Guangzhou (China). Genomic DNA was extracted from fresh young leaves combing the modified CTAB method [60] and the sequencing libraries of accessions were constructed based on instructions (Illumina). DNA from each accession included 15 plants with three biological replications. The libraries with insert sizes of ~500 bp paired-end reads were sequenced on the Illumina Hiseq4000 platform with 150 bp read length. We first made reads of accessions map to the chieh-qua ‘A36’ reference genome based on the bwa with the default parameters. For variant calling, the Genome Analysis Toolkit (version v3.2–2-gec30cee) [61] was used with the SNP detection procedure.

### Phylogenetic and population analyses

All total SNPs were used to build a neighbor-joining tree employing iqtree2 with 1000 bootstraps. The Plink [62] and Admixture (v1.3.0) were used to analyse principal component analysis (PCA) and population structure. Nucleotide diversity (Π) and population differentiation index (Fst) were calculated by vcftools (v0.1.16) using all SNPs with a window size of 200 Kb and a step size of 20 Kb. We conducted an investigation into the number (K) of ancestral clusters, ranging from 2 to 8, and inferred a phylogeny at the population level for all groups used of the maximum likelihood approach TreeMix [63]. To ensure the reliability of our analysis, samples with a genotype missing rate exceeding 20% were excluded. Additionally, SNPs with an imputation info score below 0.8, a minor allele frequency (MAF) below 5%, and a significant deviation (*P* < 10e^−4^) from Hardy–Weinberg equilibrium (HWE) were eliminated.

### GWAS

In 2020 and 2021, phenotypic data on the ratio of female flowers from a collection of chieh-qua 129 cultivars were collected. The female flower ratio was the number of female flowers occupying the total flowers in the first 30 nodes of the main stem. For each accession, we carefully measured 15 plants with three biological replications to ensure accuracy. To ensure the reliability of our analysis, we utilized SNPs with MAF greater than 5% to represent the genotypic information for each GWAS study. The GWAS analyses were conducted using GEMMA (version 0.98.1) with a mixed linear model (MLM) and the FaST-LMM program (version 2.07), taking into consideration SNPs with MAF ≥ 0.05 and a missing rate ≤ 0.4 [64]. Additionally, to minimize the occurrence of false positive results in our GWAS, we accounted for population structure by employing a kinship matrix estimated through PLINK [65]. The threshold for genome-wide significance was set at 1e^−6^, ensuring the robustness of our findings.

### BSA-seq

We performed the whole-genome re-sequencing and variant calling from the subgynoecious line JG1 and monoecious line JG128 based on the A36 genome. Two DNA pools (subgynoecy-related pool and monoecy-associated pool) were constructed and sequenced using the mixed equal amounts of DNA from 30 subgynoecious and 30 monoecious plants in the JG1 × JG128 F_2_ population for use. According to the SNPs generated from the JG1, JG129, and two DNA pools, we used the SNP index and the Euclidean distance (ED) algorithms [66] for BSA analysis. Variant calling was executed on all samples using GATK’s UnifiedGenotyper [61] and single-nucleotide polymorphisms (SNPs) and Indels were meticulously filtered using GATK’s Variant Filtration, adhering to stringent criteria (–Window 4, −filter ‘QD < 4.0||FS > 60.0|| MQ < 40.0’, −G_filter GQ < 20). To identify the precise physical locations of each variant, ANNOVAR [67], a software tool renowned for its alignment and annotation capabilities, was employed. Subsequently, thorough analysis of the calculated SNP index and delta (SNP index) [68] enabled the identification of potential regions associated with subgynoecy in chieh-qua.

### Molecular markers linkage analysis

To genotype each plant within the JG1 × JG128 F_2_ population (*n* = 200), we applied several SSR and Indel molecular markers to further fine map the target gene. The primer design was carried out using the Primer Premier 5 (Premier Biosoft, Palo Alto, CA, USA). All the newly developed markers were firstly screened for polymorphism between JG1and JG128. The primer information is listed in Table S11 (see online supplementary material).

### qRT-PCR assay

In order to know the candidate genes expression, we performed qRT-PCR assay using Eastep qPCR Master Mix (Promega, Beijing, China) based on protocols using a CFX384 Real-Time System (Bio-Rad, CA, USA). The shoot apex tissues were collected and sampled when seedlings had grown up to the four-leaf stage. The *CqUBQ* gene (*BhiCQ1080004508*) of chieh-qua was the internal control gene [28]. Relative genes expression levels were calculated via the 2^-ΔΔ^Ct method [69]. Three biological replicates were performed, and three technical repeats were performed for each biological replicate. All the primers sequences used in the study are listed in Table S12 (see online supplementary material).

## Acknowledgements

This work was funded by the National Natural Science Foundation of China (32002038), Special Fund for Scientific Innovation Strategy-construction of High-Level Academy of Agricultural Science (R2021PY-QF008), Agricultural Competitive Industry Discipline Team Building Project of Guangdong Academy of Agricultural Sciences (202114TD, 202103TD). The authors also appreciate Yueqin Heng from South China Agricultural University and Shulin Liu from Institute of Genetics and Developmental Biology, Chinese Academy of Sciences for their help in polishing the language of manuscript.

## Author contributions

M.W., S.Y., and H.L. designed the experiment and managed the project. Z.C., B.J., D.X., L.C., and S.S. collected samples, extracted genetic materials, and performed the experiments. K.W. and H.L. performed the genome assembly, annotation, and data analysis. M.W. and Q.P. performed population and agronomic trait investigation. M.W. wrote the manuscript. H.L., S.Y., and Q.P. revised the manuscript.

## Data availability

The data used to support the study results are included within the article and all the original sequencing data is uploading in the Central Authentication Service (PRJCA022068).

## Conflict of interest statement

The authors declare no conflict of interest.

## Supplementary data

Supplementary data is available at *Horticulture Research* online.

## Supplementary Material

Web_Material_uhae113
