# Correction to: Chromosome-level genome assembly and population genomics reveals crucial selection for subgynoecy development in chieh-qua

**DOI:** 10.1093/hr/uhae246

**Published:** 2024-04-22

This is a correction to: Min Wang, Zhenqiang Cao, Biao Jiang, Kejian Wang, Dasen Xie, Lin Chen, Shaoqi Shi, Songguang Yang, Hongwei Lu, Qingwu Peng, Chromosome-level genome assembly and population genomics reveals crucial selection for subgynoecy development in chieh-qua, *Horticulture Research*, Volume 11, Issue 6, June 2024, uhae113, https://doi.org/10.1093/hr/uhae113.

In the originally published version of this manuscript, the affiliation listed for Kejian Wang and Hongwei Lu was incomplete. This error has been corrected.

