# Supplementary material for: Chromosome-level genome assembly and population genomics reveals crucial selection for subgynoecy development in chieh-qua
Source: Hortic Res. 2024 Apr 22;11(6):uhae113. doi: 10.1093/hr/uhae113 (PMC11186066; doi:10.1093/hr/uhae113)
Supplement: Web_Material_uhae113 [file web_material_uhae113.zip › Supplemental Table1-5,9-12.doc]

**Supplemental Table 1 Statistics of genome repetitive sequence**

| **Type** | **Chieh-qua** | |
| --- | --- | --- |
|  | Length（bp） | Percentage of Genome（%） |
| **Class I: Retrotransposon** | 539390874 | 56.58 |
| LTR-Retrotransposon | 532958926 | 55.90 |
| LTR/Gypsy | 175932878 | 18.45 |
| LTR/Copia | 337707895 | 35.42 |
| Other | 19318153 | 2.03 |
| Non-LTR Retrotransposon | 6431948 | 0.67 |
| SINE | 15335 | 0.00 |
| LINE | 6431948 | 0.67 |
| **Class II: DNA Transposon** | 25442428 | 2.67 |
| EnSpm/CACTA | 6579819 | 0.69 |
| hAT | 2458245 | 0.26 |
| Harbinger | 134616 | 0.01 |
| MuDR | 13230436 | 1.39 |
| Helitron | 75932 | 0.01 |
| Unclassified DNA transposon | 2963380 | 0.31 |
| Tandem repeat | 24688932 | 2.59 |
| **Unclassified** | 31013494 | 3.25 |
| **Total content** | 623800155 | 65.43 |

**Supplemental Table 2 Genes characteristics statistics**

| **Types** | **HC** |
| --- | --- |
| Gene number | 34082 |
| Max gene length | 135144 |
| Min gene length | 150 |
| Gene length* | 1501/3020 |
| mRNA length* | 630/931 |
| Max cds length | 14571 |
| CDS length* | 630/931 |
| Protein length* | 209/309 |
| Exon length* | 142/227 |
| Intron length* | 252/672 |
| Exon number* | 2.0/4.1 |
| * represents median and average. |  |

**Supplemental Table 3 Statistics of genes functional annotation**

| **Chieh-qua** | **Number** | **Percent (%)** |
| --- | --- | --- |
| InterPro | 18327 | 53.8 |
| GO | 13618 | 40.0 |
| Pathway | 5856 | 17.2 |
| Pfam | 17520 | 51.4 |
| Annotated | 24593 | 72.2 |
| Unannotated | 9489 | 27.8 |
| Total | 34082 | 100.00 |

**Supplemental Table 4 Statistics of gene family clustering in chieh-qua.**

| **Item** | **Number** | **Percentage (%)** |
| --- | --- | --- |
| Genes | 34,082 | - |
| Genes in orthogroups | 29,300 | 86 |
| Unassigned gene | 4,782 | 14 |
| Orthogroups | 18,295 | 48.7 |
| Species-specific orthogroups | 967 | 5.89 |
| Genes in species-specific orthogroups | 5,878 | 17.2 |

**Supplemental Table 5 BUSCO analysis of genome annotation**

| **Parameter** | **Value** |
| --- | --- |
| Complete BUSCOs | 94.9% (1533) |
| Complete and single-copy BUSCOs | 94.2%  (1521) |
| Complete and duplicated BUSCOs | 0.7% (12) |
| Fragmented BUSCOs | 2.5% (40) |
| Missing BUSCOs | 2.6% (41) |
| Total lineage BUSCOs | 1614 |

**Supplemental Table 9 Analysis of subgynoecious trait in two seasons.**

| Season | SamNum | Mean | Sd | Median | Min | Max | Range | Skew | Kurtosis | Se |
| --- | --- | --- | --- | --- | --- | --- | --- | --- | --- | --- |
| Autumn 2021 | 124 | 0.23 | 0.12 | 0.19 | 0.08 | 0.76 | 0.68 | 1.66 | 2.94 | 0.011 |
| Spring 2022 | 115 | 0.27 | 0.16 | 0.22 | 0.1 | 0.84 | 0.74 | 1.94 | 3.36 | 0.015 |

**Supplemental Table 10 Analysis of raw data in the BSA sequence.**

| Sample | Sample Renamed | Clean_Data(bp) | Q20(%) | Total Read | Total Mapped |
| --- | --- | --- | --- | --- | --- |
| A4 | JG1 | 25486032000 | 24634167767(96.66%) | 167529006 | 166111566-99.15% |
| Shisen | JG128 | 27349979700 | 26449661111(96.71%) | 179107894 | 177978964-99.37% |
| F | subgynoecy-related pool | 30810701700 | 29745874609(96.54%) | 201703862 | 200684774-99.49% |
| M | monoecy-associated pool | 32593464300 | 31468709469(96.55%) | 213442458 | 212251042-99.44% |

**Supplementary Table 11 Primers used for gene mapping.**

| Primers | Forward | Reverse |
| --- | --- | --- |
| SSR04 | TCGAACTGGAACGACAATTGA | GGTGAGGGTGATGAGTGCAA |
| SSR08 | TGCTGAAAGCTGCAGCAAAG | TAGCTGTTTGGGTTGGGAGC |
| SSR056 | CTCGCCCTCCTTCTCTCTCT | ACACGCACGAGGAATAAGCA |
| Indel-3 | TGGCAATTGTAGTGCCAATATATG | GCTAAGCTTGTCAGCGATAGA |

**Supplementary Table 12 Primers sequences for qRT-PCR assay.**

| **Genes** | **Forward primer** | **Reverse primer** |
| --- | --- | --- |
| *BhiCQ0880026440* | CCGCCTCCATCCCTAAATCC | TCGCCGGAAAATCAAAGGGA |
| *BhiCQ0880026441* | ATTCTCCGCTTCGATTGGGG | ACAGACTCTCAAGGCGTTCG |
| *BhiCQ0880026442* | ACTGAACGGTAGGCTGAACA | CTGGAAGGGAGCAACCTAGC |
| *BhiCQ0880026443* | TCCGGCAAAGCTTTTGTGTG | TTCATGGGCCTTGGGCTTAG |
| *BhiCQ0880026448* | ACGTCGGAAACTGCCATTCT | CTGAACTTAGCGGCGGAAGA |
| *BhiCQ0880026447* | CTCGTTCTCGAGCTCCACTC | TCCTGCGCCACTTTTCTCAT |
| *BhiCQ0880026446* | CGTAAGGCTAGCGTGGATGT | TACCCACTGGAAGCAGAAGC |
| *BhiCQ0880026445* | GCTTCCAGACCCACTCTCAC | ACGACGGGGACTTTTCTGTC |
| *BhiCQ0880026449* | AGTTGAGGCCCGTCGAAATC | GCCATCTTCCTTTCCCTTCCAT |
| *BhiCQ0880026444* | ACACCCTGAAGAAGGAAGCG | TGTACCTATGCACGCTGACC |
| *CqUBQ* | CCTAACTGGGAAGACGAT | CAAGACCAAGTGAAGGGT |
